# Circulating Monocyte Subsets and Transcatheter Aortic Valve Replacement

**DOI:** 10.3390/ijms23105303

**Published:** 2022-05-10

**Authors:** Fanny Lassalle, Mickael Rosa, Bart Staels, Eric Van Belle, Sophie Susen, Annabelle Dupont

**Affiliations:** Inserm, CHU Lille, Institut Pasteur de Lille, University Lille, U1011-EGID, F-59000 Lille, France; fanny.lassalle@chu-lille.fr (F.L.); mickael.rosa@univ-lille.fr (M.R.); bart.staels@pasteur-lille.fr (B.S.); eric.vanbelle@chu-lille.fr (E.V.B.); sophie.susen@chu-lille.fr (S.S.)

**Keywords:** transcatheter aortic valve replacement, aortic valve stenosis, monocytes, monocyte subsets, inflammation, thromboinflammation, shear stress

## Abstract

Transcatheter aortic valve replacement (TAVR), as an alternative to open heart surgery, has revolutionized the treatment of severe aortic valve stenosis (AVS), the most common valvular disorder in the elderly. AVS is now considered a form of atherosclerosis and, like the latter, partly of inflammatory origin. Patients with high-grade AVS have a highly disturbed blood flow associated with high levels of shear stress. The immediate reopening of the valve during TAVR leads to a sudden restoration of a normal blood flow hemodynamic. Despite its good prognosis for patients, TAVR remains associated with bleeding or thrombotic postprocedural complications, involving mechanisms that are still poorly understood. Many studies report the close link between blood coagulation and inflammation, termed thromboinflammation, including monocytes as a major actor. The TAVR procedure represents a unique opportunity to study the influence of shear stress on human monocytes, key mediators of inflammation and hemostasis processes. The purpose of this study was to conduct a review of the literature to provide a comprehensive overview of the impact of TAVR on monocyte phenotype and subset repartition and the association of these parameters with the clinical outcomes of patients with severe AVS who underwent TAVR.

## 1. Introduction

Aortic valve stenosis (AVS) represents the most common heart valve disease in the elderly and is associated with significant morbidity and mortality when left untreated [1]. This chronic inflammatory disease is characterized by a high abundance of monocyte-derived macrophages infiltrated in the aortic valve tissue [2], promoting fibrosis and ultimately calcification of valve leaflets leading to a narrowing of the aortic valve opening, high shear stress conditions, a decrease of the arterial pulsatility and an increase of the valvular rigidity [3,4,5,6]. 

Today, surgical or transcatheter aortic valve replacement (TAVR) is the only available treatment option in patients with severe AVS [7]. In less than 20 years, TAVR has rapidly emerged as the preferred procedure for inoperable and high-surgical risk patients and recent recommendations propose to expand its indications to patients with lower-surgical risk [8,9]. However, the TAVR procedure remains associated with significant bleeding or thrombotic complications affecting short- or long-term morbidity and mortality [10]. The exact cellular and molecular mechanisms involved in these complications are still unclear and even if some biomarkers have been proposed to predict patient outcomes after TAVR, they showed limited performance and studies are still needed [11]. Furthermore, through the immediate restoration of the blood flow and reduction of the shear stress, TAVR represents a unique model for studying the impact of the blood flow pulsatility on hemostasis components and circulating blood cells, especially monocytes, the largest cells of the blood which play a key role in inflammation and hemostasis processes, linking innate and adaptive immunity. 

The historical view of the biology of monocytes has been overhauled in recent times, including their phenotypic and functional heterogeneity as well as their high sensibility to changes in their environment [12,13]. Moreover, specific monocyte subsets have already been associated with cardiovascular diseases and were shown to possess prognostic value in this context [14,15]. 

The aim of this review is to highlight recent advances regarding the effects of TAVR on monocyte functions and subset repartition as well as the prognostic value of these parameters on clinical outcomes in patients with severe AVS undergoing TAVR. To our knowledge, this is the first review of the literature on the relationship between circulating monocyte subset distribution and phenotype in the context of TAVR in patients with severe AVS. Given the important prevalence of this pathology and the significant rate of complications occurring after TAVR, this review aims to help with the prediction and understanding of these complications. At the same time, therapeutic strategies that modulate monocyte phenotype, for example, to reduce their recruitment to atherosclerotic plaques, have recently been proposed to be efficient on atherosclerosis in both animal models and a few clinical experiments [16]. An increased understanding of monocyte phenotypes and their role in TAVR complications is necessary for the development of new therapeutic approaches and is very important, since, to date, no pharmacological therapy has been shown to improve the outcomes of these patients.

## 2. Physiopathology of Aortic Valve Stenosis

AVS is the most prevalent valve disease in the elderly with an estimated prevalence from 2 to 7% in the population aged 65 years or over worldwide [17,18]. This prevalence will increase in the next decades due to an ageing population. AVS is defined by a narrowing of the aortic valve, caused by stiffening of the three valve leaflets, restricting its ability to open normally. Calcific aortic valve disease begins with an asymptomatic phase named aortic sclerosis, characterized by a moderate thickening of the aortic valve, an infiltration of some inflammatory blood cells such as monocytes and calcium deposits but without significant blood flow obstruction. The prevalence of aortic sclerosis is estimated to be 20 to 30% in people over 65 years old and almost 50% in people aged over 85 years old in developed countries [18,19]. The evolution from aortic sclerosis to AVS is slow and concerns around 1.9% of patients per year. 

AVS was previously regarded as an age-related degenerative process caused by passive calcium deposition in the valve. However, recent data challenged this concept and the valve calcification is now understood as an active inflammatory process developing through an initial endothelial injury induced by high shear stress, leading to infiltration of a large number of monocytes in the aortic valve tissue, where they differentiate into macrophages able to oxidize lipids and secrete inflammatory cytokines, chemokines and growth factors, such as tumor necrosis factor α (TNF-α), interleukin-1β (IL-1β), IL-6, transforming growth factor β1 (TGF-β1), vascular endothelial growth factor (VEGF) and matrix metalloproteinase (MMPs), leading to matrix remodeling and neovascularization [1,5,20]. Moreover, via the secretion of TGF-β1 and other inflammatory cytokines and the release of extracellular microvesicles, macrophages promote valvular interstitial cell differentiation into an osteoblastic phenotype leading to the mineralization of the extracellular matrix, and formation of calcium nodules [21,22]. Eventually, aortic cusps are calcified and the valve is stenosed, obstructing the ejection of the blood from the left ventricle to the aorta, leading to cardiac symptomatology. 

## 3. Impact of Aortic Valve Stenosis on Hemodynamic Features of Blood Flow

Cardiac valves are dynamic and closely interact with the surrounding hemodynamic and mechanical environment. In normal conditions, the aortic valve opens and closes with every heartbeat, maintaining the blood velocity and pressure gradient between the aorta and the left ventricle, experiencing the maximum shear stress during the peak systole [23]. The incomplete opening of the valve occurring during AVS produces an obstruction to the blood flow with a decrease of the arterial pulsatility and an increase of the valvular rigidity. The disturbed and turbulent blood flow leads to high shear stress impacting vascular endothelial cells and circulating blood cells [24,25,26].

These hemodynamic disorders also increase Von Willebrand factor (VWF) susceptibility to proteolytic cleavage by its main protease, a disintegrin and metalloproteinase with a thrombospondin type 1 motif, member 13 (ADAMTS13) [27,28]. VWF is a multimeric plasma glycoprotein which plays a crucial role in primary hemostasis by mediating blood platelet adhesion to the damaged vessel wall and subsequently platelet aggregation. It circulates in the plasma in an inactive globular conformation [29], but under high shear stress conditions, the protein unfolds and changes into an extended chain conformation, with the unmasking of its platelet binding sites leading to platelet adhesion and aggregation. In AVS, the high fluid shear stress promotes VWF unfolding and the prolonged exposure of the cleavage site by ADAMTS13 [30] induces an enhanced proteolysis. The loss of the VWF high molecular weight multimers resulting from this proteolysis by ADAMTS13 leads to acquired Von Willebrand syndrome [31] with potential mucocutaneous bleeding complications due to gastrointestinal angiodysplasia, known as Heyde’s syndrome [32,33]. These AVS consequences are resolved after aortic valve replacement [34,35,36]. 

## 4. Transcatheter Aortic Valve Replacement and Complications

Symptomatic severe AVS is associated with high mortality rates, up to 50% within one year, when left untreated [37]. No pharmacological treatment has been proven to delay the progression of AVS or to improve survival. Until recently, surgical valve replacement was the only therapeutic option. However, many older patients with severe AVS were contraindicated for open-heart surgery to replace stenosed aortic valves. First performed in 2002, TAVR has emerged as an alternative option for this group of patients and, recently, North American and European AVS treatment guidelines expanded its indications to younger patients with lower surgical risk [8,9]. 

TAVR is a minimally invasive procedure that consists of the implantation and deployment of a new biological valve through the femoral artery into the stenosed valve with a stent. The restoration of the valve opening and thus of the blood flow leads to an instantaneous normalization of the shear stress. The replacement of the stenosed valve also allows the immediate correction of the VWF alterations [31] with the restoration of the high molecular weight multimers 5 minutes after the new valve deployment [38]. 

However, despite a clear improvement in the patient’s prognosis and quality of life, TAVR remains associated with significant bleeding or thrombotic postprocedural complications and the management of patients after TAVR needs to be optimized. The major preoccupation is the risk of cerebral vascular accident that occurs in 3.5% of cases in the 30 days after TAVR [39,40] and 7% of cases within the first year after TAVR [41]. The occurrence of these strokes can also be asymptomatic but responsible for a premature cognitive decline [42]. Thus, silent strokes were detected by magnetic resonance imaging, performed a few days after the procedure, in more than three-quarters of the patients [43]. Short-term bleeding complications can also appear after TAVR, such as gastrointestinal bleeding (30% within 2 months) [44,45] or cerebral microbleeds (10% within 30 days) [39]. On the long-term complications, structural degeneration of the valve (tear, calcification or fibrosis) is observed in 5% of cases within 5 years [46] and prosthesis thrombosis in 1% within 5 years [47]. Finally, the mortality from cardiac causes is estimated at 30% at 5 years after TAVR [48]. 

## 5. Markers for Predicting Post-TAVR Complications 

For TAVR perioperative risk assessment, scores such as the Society of Thoracic Surgeons (STS) risk-score for predicting mortality and the European system for cardiac operative risk evaluation (EuroSCORE II), based on clinical parameters, are routinely used [49]. However, these risk scores assess surgical risk and present mediocre performance in predicting outcomes after TAVR [50]. Additional parameters or biomarkers predicting outcomes after TAVR are needed. They will also allow the identification of patients who will benefit most from the TAVR procedure, as the indications for TAVR continue to expand. 

As a systemic inflammation response occurs within 48 h after TAVR procedure in approximately 50% of patients in the absence of clinical infection and has been associated with adverse outcomes in TAVR patients, some teams tried to predict the short- and long-term TAVR complications (stroke, myocardial infarction, mortality) by measuring circulating biomarkers related to inflammation [51]. Thus, some studies demonstrated that increased pre-TAVR C-reactive protein (CRP) was independently associated with increased mortality in patients who underwent TAVR [52,53,54,55]. Recently, Navani et al. [56] focused on the platelet-to-lymphocyte ratio (PLR), an inflammatory biomarker associated with poor prognosis in patients with acute coronary syndrome [57] and did not observe an association of this biomarker, evaluated before TAVR, with the occurrence of a major adverse cardiac event (MACE), 30 days after TAVR, in a cohort of 470 patients; whereas Condado et al. observed a positive association between preprocedure PLR and MACE 30 days after TAVR in a group of 520 patient [58] and Tosu el al. [59] demonstrated in a cohort of 100 patients that an elevated preprocedure PLR was predictive of adverse outcomes (vascular complications, stroke and mortality) at 6 months after TAVR. These conflicting results may be explained by differences in the study population concerning especially the procedural risk and in the cut-off values used for PLR. Condado et al. also observed an association between elevated baseline neutrophil-to-lymphocyte ratio (NLR) and the occurrence of adverse outcomes within 30 days after TAVR; and Khalil et al. reported that NLR predicts all-cause mortality, MACE and heart failure hospitalization one year after TAVR [60]. Other studies focused on two inflammatory biomarkers, the growth differentiation factor (GDF)-15 and the soluble suppression of tumorigenicity (ST)2 (an IL-1 receptor) and reported that high levels pre-TAVR of these markers were associated with poor postprocedure outcomes [61,62,63]. Finally, these data suggest that circulating biomarkers, in addition to clinical risk scores, might help to predict complications of TAVR. However, large randomized studies are needed to clarify the utility of these biomarkers to predict outcomes of post-TAVR patients or guide clinical care [64].

## 6. Human Monocyte Heterogeneity

Monocytes are known for their very important role in tissue homeostasis and the innate immune system. Originating from bone marrow, they continuously enter the blood circulation, where they constitute 8 to 10% of the total leukocyte population in humans. During infection or damage, they are recruited into tissues where they rapidly differentiate into macrophages or dendritic cells to exert their role in inflammation, immune defense, phagocytosis or tissue repair [65]. 

Human monocytes are now classified into three functionally different subsets, based on the expression of superficial cluster differentiation CD14 (a cell co-receptor for lipopolysaccharide) and CD16 (the low-affinity Fc receptor III for IgG), with different phenotypes and functions in homeostasis and diseases [14,66,67] (Table 1). 

Classical monocytes (CD14++ CD16−) are generally short-lived cells surviving for only one day. They are rapidly mobilized into infected or injured sites and are involved in diverse functions such as phagocytosis, infection control, inflammation regulation and tissue repair. Among the three monocyte subsets, they present the greatest migration capacity in tissues [68]. 

Non-classical monocytes (CD14+ CD16++) patrol endothelial cell integrity, clear dying endothelial cells, and protect vessel health. They survive for 7 days in humans and can be recruited into sites of vascular injury or infection or enter into the areas of inflammation, such as atherosclerotic plaques [69]. 

A third monocyte subset has also been described. They are referred to as intermediate monocytes (CD14++ CD16+), which are variously described as closely resembling either classical or non-classical monocytes. They express a high degree of MHC class II gene presentation, conferring them a predominant role in inducing T-cell proliferation and stimulation and are recruited at a later stage of inflammation. The classical (CD16−) and intermediate/non-classical (CD16+) monocytes represent 80–90% and 10–15% of total monocytes, respectively. 

Although a large number of studies have focused on the differentiation of human monocyte subsets, the mechanisms regulating their recruitment into tissues and their functional and dynamic role in inflammation and immunity, there are still many aspects that need to be clarified. In this way, Cormican and Griffin [70], in a recent review about the gene expression analysis of monocyte subsets performed in the literature, pointed out conflicting results. The biggest inconsistencies remain in the production of proinflammatory cytokines by the different monocyte subsets. While Cros et al. [71] reported that classical monocytes produce high levels of reactive oxygen species (ROS) and intermediate monocytes have the highest production of TNFα and Il-1β but do not produce ROS, Wong et al. [72] showed that TNFα and Il-1β were essentially produced by non-classical monocytes with a low production of all cytokines for intermediate monocytes. Finally, Zawada et al. [73] attest that classical monocytes have the lowest production of ROS, produced the most by intermediate monocytes. It should be noted that the lack of standardization in the flow cytometry protocols for gating the different monocyte subsets can contribute to these discrepancies among studies. In any case, monocyte differentiation in these subsets seems to be tightly regulated, with the mechanisms still poorly understood. 

Many studies report the close link between blood coagulation and the innate immune system, termed “thromboinflammation” or “immunothrombosis”. Indeed, activated monocytes express on their surface a tissue factor [74] that has an essential role in the coagulation cascade, by activating factor VII which initiates the coagulation in vivo, leading to the release of thrombin, able to convert fibrinogen into fibrin; to activate coagulation factor XIII, important for stabilizing the fibrin clot; to amplify the coagulation process by activating cofactors V and VIII and factor XI and to activate platelets. Moreover, recent studies have shown that monocytes were able to express coagulation factor XIII in response to stimulation by proinflammatory cytokines [75] and to secrete tissue-type plasminogen activator, a serine protease that converts plasminogen into plasmin leading to the degradation of fibrin clots, playing a key role in fibrinolysis [76]. Monocytes can be activated by pathogen-associated molecular patterns (PAMPs) or damage-associated molecular patterns (DAMPs) and release tissue factor-bearing microvesicles [77]. Platelets and neutrophils also have a major role in immunothrombosis [78,79]. Many studies showed that an aberrant immunothrombosis process could contribute to thrombus formation in inflammatory diseases such as atherosclerosis [80]. In the AVS context, this process could be involved in disease progression and the hemostasis complications observed after TAVR.

## 7. Monocyte Subsets and Cardiovascular Diseases

Variations in the repartition of the monocyte subsets have been reported several times in diverse conditions, either as a protective mechanism or as taking part in the pathological process, such as in infections, cancers, autoimmune or inflammatory diseases [14], although the finer details of their involvement are not yet fully understood. Interestingly, the repartition of the circulating monocyte subsets has been identified as a predictive prognosis marker in various cardiovascular diseases caused by atherosclerosis such as coronary artery disease, stroke or peripheral arterial disease [12,81]. These studies frequently reported associations between an increase of intermediate monocyte levels and the severity or complications of the diseases [81,82,83,84]. For example, in a prospective cohort study of 951 subjects referred for elective coronary angiography, Rogacev et al. [85] showed that a higher level of intermediate monocytes at the inclusion was predictive of any adverse cardiovascular events (cardiovascular death, acute myocardial infarction or non-hemorrhagic stroke), after adjustment for confounders such as age, sex, diabetes, hypertension, smoking, high-density-lipoprotein cholesterol, CRP and total leukocyte count, with a mean follow-up of 3 years. Moreover, some studies highlighted that elevated intermediate monocyte levels play a key role in the growth and stability of atherosclerotic lesions [86,87]. 

## 8. Monocyte Subsets and Aortic Valve Stenosis

While data accumulate on the key role of monocytes/macrophages in AVS and the similarity in many aspects of AVS and atherosclerosis, very few studies have reported the circulating levels of total monocyte and of monocyte subsets in AVS patients. Shimoni et al. [88] compared a cohort of 54 patients with significant AVS (10 with moderate and 44 with severe AVS) to 33 patients with similar cardiovascular risk factors and no valvular disease. They observed that patients with AVS had increased levels of total circulating monocytes compared to controls with an inverse correlation between monocyte level and aortic valve area. Similarly, Efe et al. [89] observed in a cohort of 178 patients with a diagnosis of AVS (111 mild-to-moderate patients and 67 severe patients) and 139 age- and gender-matched without AVS controls, higher monocyte levels in severe AVS patients compared to mild-to-moderate patients and higher monocyte levels in mild-to-moderate AVS patients compared to controls. Moreover, they observed that the lymphocyte to monocyte ratio was lower in the group with severe AVS than in the group with mild-to-moderate AVS and lower in this group than in the control group; and that the lymphocyte to monocyte ratio was independently related to the severity of AVS (mean gradient). In both studies, they did not analyze the monocyte subsets. Hewing et al. [90] showed, in a cohort of 100 AVS patients compared to AVS free controls, that absolute levels of circulating intermediate monocytes were increased in severe AVS while absolute levels of circulating classical and non-classical monocyte subsets did not differ between both groups. Interestingly, the difference of intermediate monocyte levels between both groups was independent of age, sex, body mass index, low-density-lipoprotein cholesterol, N-terminal pro-B-type natriuretic peptide, New York Heart Association (NYHA) functional class and creatinine levels. 

## 9. Monocyte Subsets and Transcatheter Aortic Valve Replacement

A few teams were interested in the variation of the monocyte subset levels before and after TAVR that may have been induced by the sudden change of hemodynamic conditions. At this time, according to the best of our knowledge, only four studies reported these variations or associated the levels of a pre- or postprocedural subset with outcome after TAVR (Table 2). First, Hewing et al. [91] compared the monocyte subsets before aortic valve replacement versus 3 and 6 months after, in a cohort of 69 patients with severe AVS (44 TAVR and 25 surgery). They observed, in both groups (TAVR and surgery), no change in monocyte counts at 3 and 6 months after aortic valve replacement compared with baseline and a decrease of absolute intermediate monocyte levels at 6 months after surgery procedure and earlier after TAVR (at 3 and 6 months.) Absolute classical and non-classical monocytes remained stable in both groups as well as inflammatory markers (CRP, IL-6, TNF-α). Then, Neuser et al. [92] compared the monocyte subsets before TAVR versus day 4 to 7 after in a cohort of 57 patients. No difference in total absolute monocytes, classical and non-classical monocyte levels was observed, whereas they reported a decline of absolute intermediate monocyte levels after TAVR. Moreover, high levels of absolute intermediate monocytes prior to TAVR were associated with worse cardiac function and lower probability to reach an improvement in NYHA functional class 3 months after TAVR. In this study, CRP increased after TAVR but was not correlated with intermediate monocyte levels at any point. More recently, Pfluecke et al. [93] compared the three monocyte subsets on the day before, 24 h and 7 days after TAVR with the mortality at 3 months in a cohort of 120 severe AVS patients. They observed that non-classical and intermediate monocyte levels were higher before TAVR in patients who died within 3 months after TAVR compared to survivors. At 24 h and 7 days after TAVR, no significant difference was observed for the three monocyte subsets between survivors and non-survivors, except for classical monocytes the day after TAVR, higher in survivors. Interestingly, the intermediate monocyte level measured before TAVR remains an independent predictor for 3-month mortality, after adjustment with age, left ventricular ejection fraction, circulating CRP and IL-8 and CD11b-expression on monocytes, marker of cell activation. In this cohort, Cybularz et al. [94] investigated the association of frailty with monocyte subsets and observed higher absolute intermediate monocyte levels in 28 frail patients compared to not-frail patients. Moreover, intermediate monocyte levels were independent predictors for post-TAVR 6 months mortality after adjustment for frailty and CRP. Finally, Hoffmann et al. [95] compared the monocyte subsets before TAVR versus immediately, 24 h and 3 days after TAVR in a cohort of 129 patients. They observed a significant elevation of classical and intermediate monocytes at 24 h followed by an elevation of non-classical monocytes 3 days after TAVR. Moreover, they reported that levels of intermediate monocytes tended to be predictive of 12 month mortality and that non-classical monocytes measured immediately after TAVR were associated with 12 month all-cause mortality, even after the exclusion of those patients dying within the first 30 days after TAVR.

It is very difficult to compare these studies as the times of study of the monocyte subset repartitions in the post-TAVR procedure as well as the follow-up periods and type of analyzed adverse events were not the same. However, all these studies reported an impact of the TAVR procedure on intermediate monocyte levels with an earlier increase in post-procedural and then a decrease under baseline a few days and months after TAVR. 

The mechanisms involved in the modulation of circulating intermediate monocyte levels during a TAVR procedure remain speculative. The TAVR procedure results in sudden changes in wall shear stress and flow turbulence associated with a proinflammatory response. It can be hypothesized that increased levels of circulating intermediate monocytes quickly after TAVR are the result of interplay between significant hemodynamic disturbances and inflammation response. To go deeper into the comprehension of the shear stress impact on monocyte function, Baratchi et al. [96] recently compared the activation status of monocytes in patients with severe AVS before TAVR, i.e., under high shear stress, and after TAVR, i.e., normal shear stress. They showed that monocytes were more activated before TAVR in comparison with 1 month after TAVR, with a higher phagocytic activity, greater adhesive capacity and oxidized low-density-lipoprotein uptake and higher monocyte expression of proinflammatory cytokines (IL-6, interferon β1, TNFα). These results were confirmed in a microfluidic system recapitulating the shear conditions observed before and after TAVR. Interestingly, they identified the mechano-sensitive calcium channel receptor Piezo-1 as an essential mediator of the shear-stress responsive mechanoreceptor in human monocytes and observed that the expression of this receptor on monocytes is downregulated after TAVR. Thus, besides its hemodynamic benefits, a TAVR procedure also induces beneficial anti-inflammatory effects. Targeting Piezo-1 with pharmacological agents to inhibit monocyte activation may constitute a new therapeutic perspective in AVS. Interestingly, in all studies on monocyte subsets and TAVR, the authors reported an association between pre- or postprocedural levels of intermediate monocytes and TAVR deleterious complications at 3, 6 or 12 months. These results are in line with previous findings of higher major cardiovascular events depending on elevated intermediate monocyte levels [81] but these observational studies cannot answer the question whether the modulation of intermediate monocyte levels after TAVR represent a causal factor for outcomes or just a consequence of the procedure itself. Additional work is needed to understand how these modulations may influence later clinical events [12]. Moreover, one of the main limitations of these studies on TAVR patients is their small sample size. All findings should be confirmed in larger and longitudinal studies before considering intermediate monocyte levels as a usual risk marker and as a possible target for therapeutics to decrease AVS progression and the rate of complications associated with TAVR procedure. Finally, it should be noted that the impact of monocyte subsets on thrombosis and bleeding complications occurring after TAVR have not yet been described, although these complications are frequent in post-TAVR patients and monocytes are key players in the process of thromboinflammation.

## 10. Transcatheter Aortic Valve Replacement, Monocytes and Clonal Hematopoiesis of Indeterminate Potential

Clonal hematopoiesis of indeterminate potential (CHIP), defined by the presence of acquired somatic mutations in hematopoietic stem cells without other hematologic abnormalities, is a novel age-related risk factor for coronary artery calcification and atherosclerotic cardiovascular morbidity and mortality, independent of traditional risk factors [97,98,99]. Mechanistically, experimental approaches based on murine models indicate that this premalignant disorder induced an accelerated atherogenesis toward a proinflammatory and profibrotic state driven by clonally derived circulating monocytes and macrophages infiltrating atherosclerotic lesions [100,101,102,103]. Gene mutations in DNA-methyltransferase 3A (*DNMT3A*) and Tet-methylcytosine dioxygenase 2 (*TET2*) are the most frequently identified variants among patients with CHIP. Mas-Peiro et al. assessed the incidence of somatic CHIP-driver mutations in these two genes in 279 patients with critical AVS undergoing TAVR and their association with circulating monocyte subset levels evaluated before TAVR and with all-cause mortality [104]. They reported the presence of somatic *DNMT3A*- and *TET2*-CHIP-driver mutations with a variant allele frequency ≥2% in a third of patients with an incidence that increases with age (from 25% in patients aged 55–79 years to 53% in those aged 90–100 years) and a three-fold increase of death risk over a median 8-month follow-up period after TAVR in patients with *DNMT3A* or *TET2* mutations compared with a group of age-matched patients without *DNMT3A* and *TET2* mutations. They also observed in a subset of *TET2*-CHIP patients, an increase of non-classical monocyte levels compared to patients without *DNMT3A* and *TET2* mutations. As non-classical monocytes are known to secrete high levels of inflammatory cytokines [105], this data supports the notion that an inflammatory mechanism may be critical to poor outcomes in TAVR. Results of this study are strengthened by another study which observed, by using single-cell RNA sequencing, a higher expression of mediators of inflammation, IL-1β, IL-6 receptor and NOD-like receptor family, pyrin domain containing 3 (NLRP3) inflammasome complex, in the monocytes from eight patients with severe AVS and *DNMT3A* or *TET2* mutations compared to age-matched healthy control participants without *DNMT3A* and *TET2* mutations [106]. As these studies were limited to *DNMT3A* and *TET2* mutations, the effects of mutations in other driver genes on monocyte subset repartition and phenotype and the outcomes of patients with severe AVS who underwent TAVR need to be investigated. 

## 11. Preventive Therapy to Reduce Inflammation in Context of Transcatheter Aortic Valve Replacement

As we know that a systemic inflammation response occurs after a TAVR procedure in approximately 50% of patients and that monocytes seem to play a key role in this response, introducing a preventive anti-inflammatory therapy targeting monocytes should be discussed. 

Recent studies provided evidence that modulating the monocyte phenotype and inflammatory processes in pre-clinical animal models of atherosclerosis could be effective in reducing lesion size, and in patients with cardiovascular diseases in reducing cardiovascular events and improving clinical outcomes [107,108]. The most accomplished example is canakinumab, a monoclonal antibody that inhibits IL-1β, a proinflammatory cytokine predominantly synthesized by monocytes and macrophages. A large clinical trial, Canakinumab Anti-inflammatory Thrombosis Outcomes Study (CANTOS) has proved the effectiveness of this antibody in reducing the risk of cardiovascular event disease, particularly in patients with elevated markers of inflammation [109,110]. Other therapeutic approaches targeting monocytes in atherosclerosis context have been proposed, for example, by blocking C-C motif chemokine receptor 2 (CCR2)/C-C motif chemokine ligand 2 (CCL2)-mediated monocyte recruitment using small-interfering RNAs (siRNAs), monoclonal antibodies, CCR2 antagonists, pharmacological inhibition and monocyte chemoattractant protein 1 (MCP-1) inhibitors [111,112]. Moreover, in a natural way, polyphenols were demonstrated to have beneficial effects on the cardiovascular system. They are mostly present in fruits and vegetables and their actions have been studied several times [113]. Anthocyanidins (present in berries, cereals, red wine or cabbage) can reduce monocyte cell adhesion to endothelial cells by decreasing the level of adhesion molecules such as vascular cell adhesion molecule 1 (VCAM-1) and inter cellular adhesion molecule-1 (ICAM-1) as well as proinflammatory mediators such as IL-6 and MCP-1 in monocytes [114]. In a more technological way, nanoparticles represent novel sources of treatment to target cardiovascular diseases [115]. Nanoparticle-mediated targeted delivery of specific drugs into circulating monocytes has been studied in a model of atherosclerosis in mice [116] and it inhibited macrophage activation and atherosclerotic plaque rupture.

Statins were proven to reduce morbidity and to improve overall survival in patients at high risk of cardiovascular mortality by reducing the levels of low density lipoprotein-cholesterol, as well as regressing or stabilizing coronary atheromatous plaques [117,118], but they did not seem to reduce cardiovascular events related to AVS [119]. It should also be noted that data on the effects of statins on blood monocyte subset repartition are scarce and controversial [120]. However, in the context of patients undergoing TAVR, statin use was associated with a reduced all-cause mortality compared with no statin use [121,122]. Sasaki et al. [123] also studied the use of anti-atherosclerotic therapy, combining simultaneously antiplatelet agents, statins and renin aldosterone system inhibitors, prescribed after TAVR and showed that patients with this anti-atherosclerotic therapy had a better 2-year-survival rate than patients without it. 

Preventive therapy could be discussed in patients who have a pre-existing proinflammatory comorbidity, which could worsen TAVR consequences. For example, the presence of diabetes is associated with poor outcome after TAVR with a higher risk of stroke [124] or 2-year mortality [125]. As we said earlier, the mechano-sensitive calcium channel receptor Piezo-1, highly expressed on the monocyte surface, has been identified as an essential mediator of shear stress and inflammation [96]. It could be a novel therapeutic target in TAVR.

## 12. Conclusions

In the end, very few teams reported the monocyte subset repartition during TAVR but they all confirmed that intermediate monocyte levels were impacted by the instantaneous change of blood flow pulsatility occurring during the TAVR procedure in patients with severe AVS, now considered as an active and chronic inflammatory disease. In addition, as previously described for other cardiovascular diseases, intermediate monocyte levels (measured before or after TAVR) seem to be associated with early mortality or worse cardiac function after TAVR. This biomarker could be used as a predictive biomarker for TAVR complications and constitute a decision-making aid in the management of the growing population of candidates for TAVR, but, before this parameter can be used in clinical practice, these data need to be reinforced. Moreover, even though intermediate monocyte level modulation has been reported in TAVR procedures and their prognostic values have been described in some publications, the question, whether intermediate monocytes represent a reflection or rather contribute to the TAVR outcomes remains essentially unanswered. Proving the causal role of intermediate monocytes in TAVR complications in patients with severe AVS requires large prospective and long-term studies with longitudinal assessments of monocyte subsets and is an essential step towards understanding the mechanisms involved in TAVR complications and to propose, in the future, therapeutic targets of interest in the AVS and TAVR context.

## Figures and Tables

**Table 1:** Main characteristics of human circulating monocyte subsets. Abbreviations: cluster differentiation (CD), interleukin (IL), tumor necrosis factor (TNF).

Monocyte Subsets	Classical Monocytes	Intermediate MONOCYTES	Non-Classical Monocytes
Schematic representation	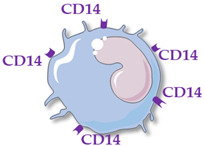	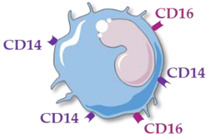	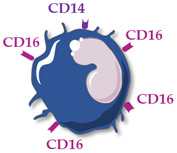
Surface receptors	CD14++ CD16−	CD14++ CD16+	CD14+ CD16++
Proportion of total monocytes	85–90%	5–10%	5–10%
Main functions	Phagocytosis, tissue repair, inflammation, reactive oxygen species production	Antigen presentation, T-cell proliferation and stimulation, reactive oxygen species production, phagocytosis	Patrolling of endothelial cell integrity, clearance of dying endothelial cells, wound healing
Cytokine production	IL-10, IL-6	TNFα, IL-1β, IL-6	TNFα, IL-1β, IL-6

**Table 2:** Evolution of the circulating monocyte subsets (absolute values, compared to pre-transcatheter aortic valve replacement (TAVR)) in patients with aortic valve stenosis underwent TAVR. Age, Society of Thoracic Surgeons (STS) score and European system for cardiac operative risk evaluation (EuroSCORE II) are presented as mean ± standard deviation or median (interquartile ranges) according to available data. *: post-TAVR; =: stable; ↗: increase; ↘: decrease. Abbreviation: New York Heart Association (NYHA).

Number; Origin of Patients	Age; Gender Proportion	STS Score (%) EuroSCORE II (%)	Time of Blood Sampling	Classical Monocytes	Intermediate Monocytes	Non-Classical Monocytes	Association with Outcomes	Reference
44; Germany, single center	80.2 ± 6.1; 50% male	2.5 (1.4–3.9); 3.6 (2.3–5.7)	Pre-TAVR				Not available	Hewing [91]
			3 months *	=	↘	=		
			6 months *	=	↘	=		
57; Germany, single center	83.3 ± 0.79; 47% male	5.97 ± 0.39; 6.71 ± 0.65	Pre-TAVR				High levels of intermediate monocytes pre-procedure associated with worse cardiac function and lower probability to reach an improvement in NYHA 3 months after TAVR	Neuser [92]
			Day 4 to 7 *	=	↘	=		
120; Germany, single center	81; 33% male	>4	Pre-TAVR	No comparison between times			High levels of intermediate monocytes pre-procedure associated with 3-month mortality	Pfluecke [93] Cybularz [94]
			24 h *					
			Day 7 *					
129; Germany, single center	83 (79–86); 76% male	3.41 (2.45–4.94) 3.31 (2.31–6.04)	Pre-TAVR				High levels of intermediate monocytes pre-procedure trended to be predictive of 12-month mortality and high levels of non-classical monocytes post-procedure associated with 12-month mortality	Hoffmann [95]
			24 h *	↗	↗	↗		
			Day 3 *	↗	↗	↗		

## Data Availability

Not applicable.
